# Anoplastie périnéale simple pour le traitement des malformations anorectales basses chez l'adulte, à propos de deux cas

**DOI:** 10.11604/pamj.2014.19.27.4846

**Published:** 2014-09-12

**Authors:** Abdelmoughit Echchaoui, Malika Benyachou, Jawad Hafidi, Nahed Fathi, Elhamid Mohammadine, Samir ELmazouz, Nour-eddine Gharib, Abdellah Abbassi

**Affiliations:** 1 Service de Chirurgie Réparatrice et Plastique, CHU Avicenne, Rabat, Maroc; 2 Service de Chirurgie Générale, CHU Avicenne, Rabat, Maroc

**Keywords:** Anoplastie périnéale, malformations anorectales, dyschésie, anovulvaire, perineal anoplasty, anorectal malformations, dyschezia, ano-vulvar

## Abstract

Les malformations anorectales chez l'adulte sont des anomalies congénitales rares du tube digestif qui prédominent chez le sexe féminin. Notre étude porte sur deux observations de malformation anorectale basses vues et traitées au stade adulte par les 2 équipes (plasticiens et viscéralistes) à l'Hôpital Avicenne à Rabat. Il s'agit d'un homme de 24 ans avec une dyschésie anale l'autre cas est une femme de 18 ans avec une malformation anovulvaire Les caractéristiques cliniques combinées avec les imageries radiologiques (lavement baryté, et la manométrie anorectale) ont confirmé qu'il s'agit d'une malfomation anorectale basse. Les deux cas sont corrigés par une reconstruction sphinctérienne, réimplantation anale avec anoplastie périnéale. Les suites opératoires étaient simples, pas de souffrance cutanée ou nécrose, avec changement de pansement gras chaque jour. Le résultat fonctionnel (la continence) était favorable pour les 2 patients. La présentation des MAR à l’âge adulte est rare, d’étiologie mal connu, elles apparaissent selon le mode sporadique. Les caractéristiques cliniques, couplées à l'imagerie (lavement baryté, IRM pelvienne), l'endoscopie et la manométrie anorectale, permettent de confirmer le diagnostic et classer ces anomalies en 3 types: basses, intermédiaires, et hautes. Les formes basses sont traités d'emblée par une réimplantation anale et anoplastie périnéale simple tels nos deux cas, elles peuvent être traités dans certains cas par un abaissement anorectale associé à une plastie V-Y permettant ainsi un emplacement anatomique correct de l'anus; alors que les formes hautes ou intermédiaires relèvent d'une chirurgie complexe avec souvent une dérivation digestive transitoire. Contrairement aux autres formes, Les formes basses ont un pronostic fonctionnel favorable.

## Introduction

Les malformations anorectales chez l'adulte regroupent des anomalies congénitales diverses allant de la simple malposition anale à l'absence totale du rectum et de l'anus. Elles prédominent chez le sexe féminin et surtout dans les pays en voie de développement. Ce sont des malformations rares, qui persistent jusqu’à l’âge adulte car elles ne sont pas détectées à la naissance ou cachées par les familles.

## Méthodes

Notre étude porte sur deux observations de malformations anorectales basses vues et traitées au stade adulte par deux équipes chirurgicales (chirurgie plastique et chirurgie générale) à l'Hôpital Avicenne à Rabat.

Patiente de 18 ans sans antécédents notables, adressée au service lors d'une consultation prénuptiale où elle rapporte la notion de défécation par voie vaginale depuis son enfance. L'examen proctologique montre un aspect anovulvaire anormal avec un orifice vaginal antérieur qui semble contenir deux orifices de tailles inégales et un orifice anale qui est séparé par un raphé membraneux fin, au toucher rectal: il y avait une hypotonie sphinctérienne et la présence d'une cloison recto-vaginale. Le lavement baryté est réalisé avec difficulté car le patient lâche la baryte.

Patient de 24 ans qui présentait depuis sa naissance une constipation d’évacuation et une incontinence anale et chez qui l'examen clinique trouve un orifice anal d'aspect anormal, rétréci et infranchissable au doigt. On a complété le diagnostic par des examens paracliniques nécessaires. La manométrie anorectale était en faveur d'une dyschinésie anorectale avec mégarectum. Le lavement baryté a montré une dilatation du rectum et du sigmoïde en amont d'un rétrécissement de la région anorectale.

## Résultats

**Figure 1:**
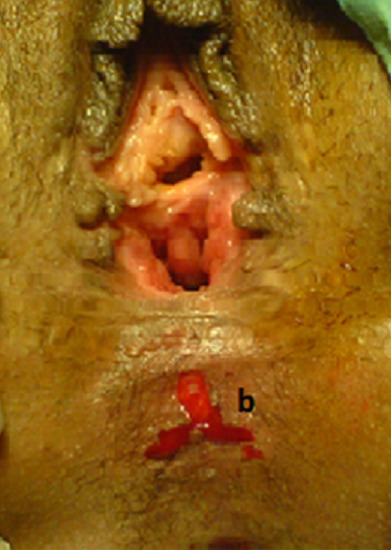
Le siège de l'incision en trèfle (b) en regard du sphincter externe après le repérage de ses contractions par des stimulations électriques

**Figure 2:**
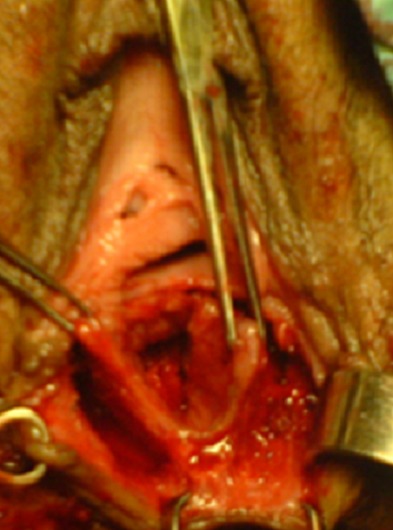
Dissection, abaissement et réimplantation du canal anal

**Figure 3:**
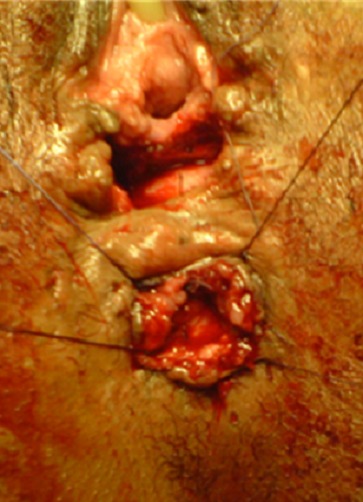
Séparation de l'anus du vagin par un pont de peau saine et anoplastie périnéale

**Figure 4:**
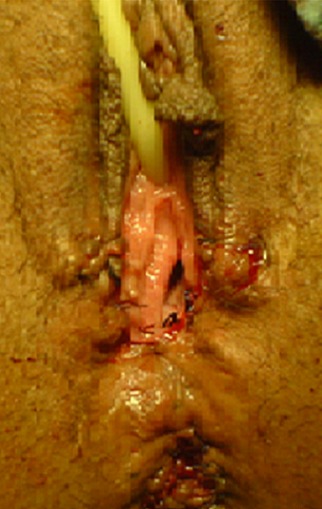
Rétablissement des structures anatomiques d'allures habituelles de la vulve et du périnée (néo anus)

Pour le premier cas sous une rachi-anesthésie, l'intervention a été faite en deux temps. Dans un premier temps, par des stimulations électriques, on a repéré l'endroit des contractions musculaires sphinctériennes et nous avons réalisé une incision en trèfle (siège de la réimplantation du canal anal) (Figure 1). Dans un 2^ème^ temps l'orifice du canal anal a été disséqué, abaissé à travers le sphincter anal externe (Figure 2), puis suturé par des points au vicryl 3/0 (Figure 3), la cloison recto vaginale a été suturée dans un second temps (Figure 4).

**Figure 5:**
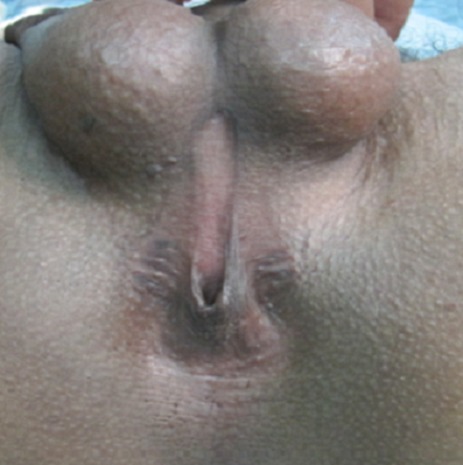
Anomalie de perforation anale (anus rétréci)

**Figure 6:**
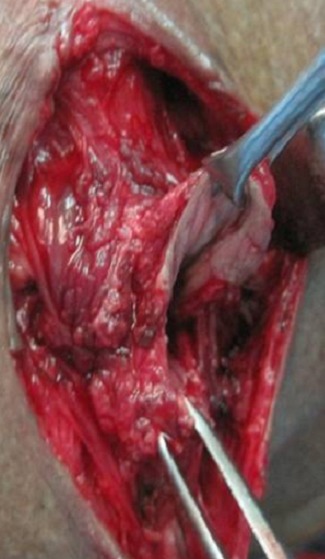
Dissection du canal anal qui est réimplanté ultérieurement

**Figure 7:**
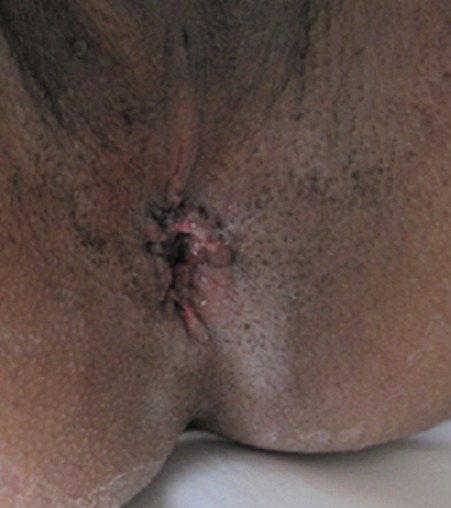
Résultat après une semaine d'intervention

Pour le deuxième cas et vu l'expérience acquise lors du premier cas, une malformation anorectale avec une imperforation anale et ectopie du canal anal a été retenue (Figure 5) et le malade a été proposé pour réimplantation anale. Sous une rachi-anesthésie, on a réalisé une Incision périanale avec dissection et individualisation des différentes structures anatomiques (fibres superficielles du sphincter externe) avec une hémostase rigoureuse (Figure 6). Dans un 1^er^ temps il fallait repérer l'emplacement habituel du sphincter externe. Puis une réimplantation anale avec une anoplastie périnéale (Figure 7).

Pour les deux cas une triple antibiothérapie (Céfriaxone, Gentamycine, Métronidazol) est commencée en post-opératoire est gardée pendant dix jours (3 j pour la Gentamycine). Les suites opératoires étaient simples, il n y avait pas de souffrance ou nécrose cutanée ni de lâchage de sutures chez les deux cas avec changement du pansement au miel chaque jour. Les deux patients ont été gardés sous alimentation parentérale pendant 5 jours puis réintroduction d'une alimentation douce. Ils ont tous deux été revus le quinzième jour pour une dilatation anale digitale. Le résultat fonctionnel (la continence sphinctérienne) était favorable chez nos deux patients.

## Discussion

En 1835 Jean Zoléma Amussat était le premier qui a suturé la paroi rectale aux bords de la peau, ce qui pourrait être considéré comme la première anoplastie [1]. L’étiologie reste floue et probablement multifactorielle, la cause génétique est à ne pas écarter [2]. La fièvre pendant le premier trimestre de la grossesse et les emplois responsables de l′exposition maternelle aux agents de nettoyage et les solvants sont des facteurs qui ont été incriminés pour engendrer ces types de malformations [3]. La présentation des MAR à l′âge adulte est rare, elles apparaissent selon le mode sporadique [4]. Les caractéristiques cliniques (constipation ou incontinence aux selles douleurs et/ou distension abdominales) [5], couplées à l'imagerie médicale (lavement baryté, IRM pelvienne), l'endoscopie et la manométrie anorectale, permettent de confirmer le diagnostic et classer ces anomalies en 3 types selon la classification de Krickenbeck [6]: basses, intermédiaires et hautes.

Les formes basses sont plus fréquentes [7] et sont traités d'emblée par une réimplantation anale et anoplastie périnéale simple suivi d'une dilatation anale progressive pour éviter la sténose [8], elles peuvent être traités par un abaissement anorectale avec une anoplastie V-Y permettant ainsi un emplacement anatomique correct de l'anus et une continence acceptable pour le patient [9]; alors que les formes hautes ou intermédiaires relèvent d'une chirurgie complexe avec souvent une dérivation digestive transitoire. Contrairement aux autres formes, Les formes basses ont un pronostic fonctionnel favorable [10, 11].

## Conclusion

Les cas de malformations anorectales détectés au stade adulte: sont des formes négligées à la naissance. Les techniques chirurgicales proposées pour la réparation sont nombreuses en fonction du type anatomique. L'anoplastie périnéale simple donne de meilleurs résultats anatomo-fonctionnels dans les formes basses. Ces malformations peuvent avoir un impact sur la vie du patient, d'où la nécessité de les dépister à la naissance et de les traiter précocement à fin d'améliorer sensiblement leur pronostic.
